# Biotechnological Insights on the Expression and Production of Antimicrobial Peptides in Plants

**DOI:** 10.3390/molecules26134032

**Published:** 2021-07-01

**Authors:** Balamurugan Shanmugaraj, Christine Joy I. Bulaon, Ashwini Malla, Waranyoo Phoolcharoen

**Affiliations:** 1 Baiya Phytopharm Co., Ltd., Bangkok 10250, Thailand; balamurugan.s@baiyaphytopharm.com (B.S.); ashwini.m@baiyaphytopharm.com (A.M.); 2 Research Unit for Plant-Produced Pharmaceuticals, Chulalongkorn University, Bangkok 10330, Thailand; 6371002633@student.chula.ac.th; 3 Department of Pharmacognosy and Pharmaceutical Botany, Faculty of Pharmaceutical Sciences, Chulalongkorn University, Bangkok 10330, Thailand

**Keywords:** antimicrobial peptides, antibiotic-resistance, biopharmaceuticals, heterologous expression, molecular farming, plant expression system, stable expression, transient expression

## Abstract

The emergence of drug-resistant pathogens poses a serious critical threat to global public health and requires immediate action. Antimicrobial peptides (AMPs) are a class of short peptides ubiquitously found in all living forms, including plants, insects, mammals, microorganisms and play a significant role in host innate immune system. These peptides are considered as promising candidates to treat microbial infections due to its distinct advantages over conventional antibiotics. Given their potent broad spectrum of antimicrobial action, several AMPs are currently being evaluated in preclinical/clinical trials. However, large quantities of highly purified AMPs are vital for basic research and clinical settings which is still a major bottleneck hindering its application. This can be overcome by genetic engineering approaches to produce sufficient amount of diverse peptides in heterologous host systems. Recently plants are considered as potential alternatives to conventional protein production systems such as microbial and mammalian platforms due to their unique advantages such as rapidity, scalability and safety. In addition, AMPs can also be utilized for development of novel approaches for plant protection thereby increasing the crop yield. Hence, in order to provide a spotlight for the expression of AMP in plants for both clinical or agricultural use, the present review presents the importance of AMPs and efforts aimed at producing recombinant AMPs in plants for molecular farming and plant protection so far.

## 1. Introduction

Various antibiotics are used for the prevention or treatment of many common diseases caused by pathogenic organisms. Microbes have the ability to circumvent the mechanism of antibiotic drugs resulting in the development of antimicrobial resistance. Many available evidences showed that the frequent use of large amounts of conventional antibiotics result in drug resistant pathogens, particularly antibiotic-resistant bacteria. The growing burden of drug-resistant infections demand for suitable antimicrobial/antibiotic alternatives. AMPs are ubiquitous short peptides that exhibit broad spectrum of potent antimicrobial efficacy and are capable of being applied to treat various microbial infections including drug-resistant ones. AMPs, also referred as peptide antibiotics have gained significant prominence as innovative antibiotics with great importance in the last few years. Several AMPs with potent antimicrobial properties against bacteria, fungi and virus have been documented. These peptides are shown to have antimicrobial activities by obstructing the functionality of biological molecules present in the cell membrane, making the microbes susceptible [1,2]. Hence, instead of conventional antibiotics, these peptides have promising opportunity to develop into antimicrobial drugs. The importance and therapeutic potential of AMPs have been reviewed in detail elsewhere [3,4,5].

AMPs are reported to exhibit a broad spectrum of antiviral, antifungal, antiparasitic, immunomodulatory and anti-angiogenic activities [6,7,8,9]. The anionic/cationic charges, amphiphilic and hydrophobic properties of AMPs were determined by their amino acid composition which in turn show selective effects on the microbes. Though most of the AMPs are cationic with substantial hydrophobic residues, anionic AMPs containing mostly acidic amino acids like aspartic acid and glutamic acid also exist [10,11]. AMPs display antimicrobial activity by a unique mode of action via targeted destruction of the bacterial membrane and/or by translocation into the cytoplasm to neutralize intracellular targets [1,12,13]. The direct activity against bacteria involves strong electrostatic interaction of positively charged AMPs with the negatively charged microbial surface [14,15]. Bacterial membranes comprise abundant number of amphiphilic lipids such as phosphatidylglycerol, cardiolipin, and phosphatidylethanolamine in their cytoplasmic membranes. The head groups of anionic phospholipids are negatively charged and bind strongly to cationic AMPs [15,16]. Moreover, teichoic acids and lipopolysaccharides in gram-positive and gram-negative bacteria, respectively, provide electronegative charge to bacteria making them highly attractive targets for AMPs [17,18]. The interaction of AMP with the microbial membrane eventually results in destroying the microbes. In contrast, mammalian cell membranes differ from microbial membranes by having rich amount of zwitterionic phospholipids such as phosphatidylethanolamine, phosphatidylcholine, and sphingomyelin in their cytoplasmic membranes [19]. These phospholipids produce neutral charge on mammalian membranes resulting in low binding efficiency of AMPs and enable protection and selectivity against mammalian cells [15,19]. Further AMPs bind with mammalian membranes via weak hydrophobic interactions and are rich in cholesterol which reduces activity of these peptides [20].

To date, more than 2000 AMPs have been identified, synthetically designed, engineered accounting from all living forms including prokaryotic and eukaryotic organisms [21]. Native AMPs can be classified according to source, activity, structure and amino acid residues wherein examples include but are not limited to defensins [22], cathelicidins [23], cecropins [24], lactoferricin [25], dermcidin [26], and anionic peptides [11]. Since their discovery, AMPs have been of great scientific interest due to their importance in human health, as natural antibiotic agents, but also due to their potential as innate immune modulators [27].

Due to its therapeutic potential against drug-resistant pathogens, AMPs are considered as next generation of antimicrobials having potential for pharmacological applications. Furthermore, certain AMPs exert a broad spectrum of activity against diseases in plant species caused by different plant pathogens including bacterial, fungal and viral pathogens [28]. Hence, they represent an innovative crop plant protection method for engineering insect or disease resistance traits for sustainable agriculture. Although AMPs are widely found in their natural form, their synthesis involves cumbersome processes that result in low yields and has so far not proven cost-effective [29,30,31,32,33]. Several features of AMP manufacturing limit the commercial development of these peptides. Chemical peptide synthesis based on solid-phase techniques is a mature technology [34] allowing the production of naturally existing or synthetic polypeptides of small size. In the context of green sustainable chemistry, to avoid the use of large amounts of organic solvents in the chemical synthesis of AMPs, water-based solid phase peptide synthesis using water to replace organic solvents has been developed, facilitated by the conversion of amino acids into water-soluble forms. However there exist a few limitations such as the requirement of additional conversion steps, high preparation costs, need of more energy and resources [35,36]. Moreover, this method also faces issue in the production of large peptides with complex post-translational modifications. Contemporary research utilizes a sophisticated approach to produce AMPs. The recombinant production of AMP in heterologous expression systems provide an opportunity for large scale production of AMPs thereby increasing their accessibility and broadening of their applications in the pharmaceutical sector. Genetic engineering strategies have been employed for production of AMPs in microbial and eukaryotic systems. Traditionally bacteria, yeast and mammalian cells were commonly used for recombinant protein production, however plant-expression systems have considerable advantages like flexibility, scalability and speed. Hence in recent decade, plants have become considered an affordable recombinant protein expression platform. Although plants have been widely utilized for producing vaccine antigens, diagnostic reagents and other high-value biopharmaceuticals in recent times, the recombinant production of AMPs in plants are very limited. Therefore, we focus in this review on the importance of AMPs, possibilities and biotechnological approaches for the expression and production of pharmaceutically important AMPs in plants and further discuss the recent progress which has been made in this direction. Overall, some of the major advances in the field with the view to provide insights on the production of AMPs in plants are presented.

## 2. AMPs for Infection and Therapy

AMPs differ from antibiotics by having broad range and rapid inhibitory activities against bacteria, fungi, parasites, viruses and in their capacity to overcome resistance in microbial populations [18,37]. Most AMPs directly target the bacterial cell membrane which results in comparable levels of action on multidrug-resistant organisms. Consequently, combined treatment with other intracellular drugs postulates no overlap in modes of action and cross-resistance effects. Thus, considering desirable potency and bactericidal ability, AMPs constitute a promising class of therapeutics for the treatment of drug-resistant infections [3].

To date, a number of AMPs are either in pre-clinical and clinical development or approved for commercial applications. Selected AMPs are listed in Table 1. Polymyxins are one of the most well-studied cyclic peptides utilized as clinically available drugs or treatment for eye infections and multidrug resistant pathogens and are also used for selective digestive tract decontamination and local skin infections [38,39]. Gramicidins are another type of cyclic anti-infective peptides used to treat wounds and local infections in the nose, eyes, and throat [40,41]. Daptomycin is one of the cyclic AMP-based drugs recently approved by the FDA for the treatment of complicated skin and skin-structure infections (cSSSI) caused by *Staphylococcus aureus* [42,43]. Meanwhile, some of the AMPs under clinical trials, including well-characterized peptides—pexiganan and omiganan—are presently being investigated for the treatment of many bacterial and fungal infections. Accordingly, pexiganan is a *Xenopus* magainin analog targeting bacteria, fungi, and antibiotic-resistant microbes. It has been assessed in phase III clinical trials and administered as a topical cream for diabetic foot ulcers [44] and further examined for cSSSI [42]. Of note, omiganan is a bovine indolicidin analog that has been evaluated in clinical trials and administered as a topical gel for catheter infections, rosacea, dermatitis, genital warts and inflammatory acne vulgaris [3,45,46]. On the contrary, a few AMPs under clinical trials such as LL-37 and PXL-01 are being evaluated for their mode of action unrelated to microbial infections. For LL-37, pre-clinical results showed its role in wound healing in mice [47,48] and wound infections in pigs [49]. In line with these reports, a phase I/II clinical trial using topical treatment with LL-37 markedly promoted wound healing rates in patients with hard-to-heal chronic leg ulcers [50] and has been recommended to regulate re-epithelialization, angiogenesis and inflammatory response [51]. For PXL01, nonclinical evidence revealed anti-adhesion properties [52] and it effectively prevented adhesion formation linked to post-abdominal surgery in rats [53]. A phase II clinical study further established the efficacy of using PXL01 in sodium hyaluronate for inhibiting post-surgical adhesions and improving post-surgical recovery of the hand [54].

Consideration of AMPs for their clinical applications requires that the underlying issues of activity, toxicity and stability be addressed to achieve progress and commercial success. An ideal AMP should display high antimicrobial activity and specificity, less toxicity towards mammalian cells, high stability and low production costs. Despite showing antimicrobial potency against the microbes, in long term use AMPs have undesirable activities by eliciting immediate immunogenic responses, systemic toxicity, hemolytic activity and other side effects in mammalian cells or in vivo animal models. Hence detailed studies are essential to assess the feasibility and safety profile of AMPs before progressing them towards practical application. The therapeutic index, which is calculated as the ratio of the hemolytic activity and antimicrobial activity of AMPs, is a widely used parameter to evaluate the specificity of AMPs against prokaryotic and eukaryotic cells. Thus, higher values of therapeutic index represent greater specificity [55]. Some AMPs have demonstrated nephrotoxicity and neurotoxicity effects [56], frequently associated with high dosages. Further poor stability of AMPs is another major limitation that critically affects their oral administration, as peptide antibiotics are characterized with low oral bioavailability owing to enzymatic degradation and poor permeability in the intestinal mucosa. Likewise, systemic administration via intravenous injection restricts applications of AMPs due to rapid degradation or rapid hepatic and liver clearance resulting in significantly reduced or short half-life [57]. Selective drug delivery methods can address some of the limitations hindering its applications *viz*., topical application of AMPs may reduce the systemic toxicity and proteolytic stability [58]. To overcome the limitations and multiple restrictive factors, many approaches have been employed to develop ideal AMPs, including multi-disciplinary strategies with computational/bioinformatic tools, biophysical experiments and biological validations which are discussed in detail elsewhere [59,60].

**Table 1:** List of few AMPs at various stages of clinical trials.

Anti-Microbial Peptide	In Vivo/Clinical/Approved	Indication	Administration	Reference
Mutacin B-Ny266 (lantibiotic)	In vivo	Multi-drug resistant bacteria infection	-	[61]
Actagardine (lantibiotic)	In vivo	Staphylococcal, enterococcal, *C. difficile* infections	-	[62]
Plectasin (defensin)	In vivo	Systemic pneumococcal and streptococcal infections	-	[63]
Planosporicin (lantibiotic)	In vivo	Staphylococcal and enterococcal infections	-	[64]
Gallidermin/Epidermin (lantibiotic)	In vivo	Acne, eczema, folliculitis, and impetigo	-	[65]
Microbisporicin (lantibiotic)	In vivo	Staphylococcal and enterococcal infections; Acne	-	[66]
Mersacidin (lantibiotic)	In vivo	Staphylococcal, enterococcal, *Clostridioides difficile* infections	-	[67]
Lacticin 3147 (lantibiotic)	In vivo	Staphylococcal and enterococcal infections; Acne	-	[68]
Salivaricin B (lantibiotic)	In vivo	Streptococcal infections (caused mainly by *S. pyogenes*) and dental caries	-	[69]
Duramycin (lantibiotic)	In vivo	Cystic fibrosis, ocular diseases, and disorders	-	[70]
Deoxyactagardine/NVB302 (lantibiotic)	In vivo	*C. difficile* infections	-	[71]
Nisin (lantibiotic)	In vivo	Staphylococcal and enterococcal infections	-	[72]
Pinensins (lantibiotic)	In vivo	Yeast/fungal infections	-	[73]
MX-226	In vivo	Catheter infections	-	[74]
PAC-113 (histatin 3)	Phase II Identifier: NCT00659971	Oral candidiasis in HIV patients	Oral (Mouthwash)	[75]
Omiganan (indolicidin)	Phase III Identifier: NCT00231153	Prevent local site catheter infection and colonization with central venous catheters	Topical	[46]
Iseganan (protegrin-1)	Phase II Identifier: NCT00118781	Ventilator-associated pneumonia	Oral (Mouthwash)	[76]
	Phase III Identifier: NCT00022373	Oral mucositis induced by chemotherapy	Oral (Mouthwash)	[77]
Pexiganan (magainin analog)	Phase III Identifier: NCT00563-394/433	Diabetic foot ulcer infections	Topical	[44]
hLF1-11 (lactoferrin)	Phase I/II Identifier: NCT00509938	Bacteraemia and fungal infection	Intravenous	[78]
CZEN-002 (α-melanocyte-stimulating hormone)	Phase IIb	Vaginal candidiasis	Vaginal gel	[79]
Novexatin (defensin)	Phase II Identifier: NCT02343627	Stubborn fungal nail infection	Topical	[45]
LL-37 (cathelicidin)	Phase I/II Identifier: NCT04098562	Hard-to-heal venous leg ulcers	Topical	[50]
PXL01 (lactoferricin)	Phase II Identifier: NCT01022242	Prevent post-operative adhesion in hands	Hydrogel applied at surgical site	[54]
Surotomycin (synthetically modified daptomycin)	Phase III Identifier: NCT01597505	Diarrhea caused by *C. difficile*	Oral	[80]
LTX-109 (synthetic antimicrobial peptidomimetic)	Phase II Identifier: NCT01803035	Skin infection, impetigo	Topical	[81]
	Phase I/II Identifier: NCT01158235	Nasal infection with *S. aureus*	Nasal	[81]
SGX942 (indolicidin)	Phase III Identifier: NCT03237325	Oral mucositis induced by radiation and/or chemotherapy	Intravenous	[82]
OP-145 (cathelicidin)	Phase I/II	Chronic otic infection	Eardrops	[83]
C16G2 (synthetic specific-directed antimicrobial peptide)	Phase II Identifier: NCT02044081	Avoid caries caused by *S. mutans*	Oral (Mouthwash)	[84]
Murepavadin (protegrin I)	Phase I Identifier: NCT03409679	Ventilator-associated pneumonia and bronchiectasis by *Pseudomonas aeruginosa*	Intravenous	[85]
DPK-060 (hybrid peptide from 2 functional domains)	Phase II Identifier: NCT01522391	Human wound infection caused by *S. aureus*	Topical	[86]
Teicoplanin (*Actinoplanes teichomyceticus* glycopeptide)	Approved	Bacterial infections	Intravenous and Intramuscular	[87]
Daptomycin (anionic peptide)	Approved	Bacterial skin infections	Intravenous	[43]
Colistin (*Bacillus polymyxa* cyclic peptide)	Approved	Multi drug-resistant gram-negative infections	Intravenous	[88]
Dalbavancin (Teicoplanin derivative lipoglycopeptide)	Approved	Acute bacterial skin and skin structure infections	Intravenous	[89]
Polymyxin (*Bacillus polymyxa* polypeptide)	Approved	Urinary tract and bloodstream infections	Ophthalmic Topical Intravenous	[38]
Enfuvirtide (biomimetic peptide)	Approved	HIV-1 infection	Subcutaneous	[90]
Telavancin (vancomycin derivative lipoglycopeptide)	Approved	Bacterial skin infections	Intravenous	[91]
Gramicidin D (*Bacillus brevis* polypeptides)	Approved	Skin and eye infections	Topical Ophthalmic	[40]
Oritavancin (vancomycin derivative lipoglycopeptide)	Approved	Bacterial skin infections	Intravenous	[92]
Bacitracin (*Bacillus licheniformis* cyclic peptide)	Approved	Skin and eye infections; wound infections	Topical	[93]
Telaprevir (antimicrobial peptidomimetic)	Approved	Hepatitis C infection	Oral	[94]
Vancomycin (*Amycolatopsis orientalis* glycopeptide)	Approved	Bacterial infections	Oral and Intravenous	[95]

## 3. Heterologous Production of AMPs

Recent advances in recombinant DNA engineering provide an insight for the economical production of AMPs in various heterologous host systems [96,97,98,99]. Furthermore, recombinant expression platforms will undoubtedly speed up the approaches for developing novel peptide therapeutics and are also helpful for the betterment of existing ones. Many expression hosts are currently available for the production of various short AMPs with varied sizes, folds and complexities. Certain factors like size, intracellular localization, secretion, protein folding and glycosylation need to be considered during the selection of a host expression system to produce AMPs. Microbial systems (bacteria and yeast) are the most widely employed as they are easy to manipulate and have rapid growth rates, multiplication times and high cell densities [100]. Bacterial species such as *Escherichia coli*, *Bacillus subtilis*, *Propionibacterium freudenreichii* were used for expression of different AMPs like adenoregulin, cecropin, crustin, defensin, hepcidin, histonin, human β defensin, lactoferrin, perinerin, thanatin and viscotoxin [101]. *E. coli* was the most prominent bacterium used to express AMPs, due to its easy growth rate, well developed recombinant methods for its manipulation and the abundant available literature on its genetic morphology and physiology [2,63]. Though many AMPs are expressed in bacteria, there are few hurdles that need to be addressed in order to achieve efficient production. The produced AMPs that have natural activity must be prevented from exerting their lethal action on the host strain. A lack of post-translational modification and the need for carrier/fusion proteins are other issues [2,63,102,103]. *Pichia pastoris* and *Saccharomyces cerevisiae* are commonly used for the expression of AMPs such as the antifungal proteins cathelicidin, enterocin, pediocin, plantaricin, and α-sarcin [101,104,105]. Nevertheless, plant systems have also been utilized for AMP production in recent decades. Tobacco is one of the most highly explored leaf-based production platforms for recombinant protein expression.

## 4. Plant Molecular Farming

The expression of AMPs in plants presents a dual role as their antimicrobial activity helps in plant protection while also meeting the demand for novel antimicrobial agents in the biopharmaceutical industry [106]. The process of utilizing plants and plant cell cultures as an effective production platform for recombinant proteins with industrial or pharmaceutical significance is called molecular farming and the protein products are often referred to as plant-made pharmaceuticals (PMPs). Plants act as remarkable hosts for producing various recombinant proteins due to their many advantages over other prokaryotic and eukaryotic expression systems. The major propitious features include the cheaper cost, high yields with the feasibility for easy scale up, simple manufacturing methods, minimizing the extensive purification and processing techniques in the case of oral vaccines [107,108,109]. The plant-made vaccines or therapeutic products can be easily stored or lyophilized for longer shelf life without the requirement of low temperatures for keeping them stable and retaining their activity [110]. Further, the pharmaceutically-relevant proteins produced in plants are considered to be safer when compared to bacterial or mammalian cells as the risk of contamination during the manufacturing processes is low and the do not present serious bio-safety threats [111]. The major advantage of this expression system is the ability to perform post-translational modifications which are likely crucial for protein folding and the biological function of AMP molecules [112,113]. The advantages and challenges of different protein production systems are summarized in Table 2.

In 1986, the recombinant human growth hormone was the first plant-derived pharmaceutically-relevant protein produced in transgenic tobacco and sunflower [115], followed by the report of functional antibody expression in transgenic tobacco plants [116]. After two decades of research, the first PMP “Elelyso” (recombinant β-glucocerebrosidase) produced in carrot suspension culture was approved by FDA in 2012 for the treatment of Gaucher’s disease. In addition, the regulatory approval of tobacco-produced HIV-neutralizing human monoclonal antibody 2G12 established the scientific, technical and regulatory framework for plant-derived recombinant proteins [117]. Recently, virus-like particle (VLP) influenza vaccine manufactured in plants has completed the phase III trial and plant-derived VLP vaccine for coronavirus disease produced in *N. benthamiana* has completed a Phase I trial [118]. Furthermore, number of PMPs are in various stages of clinical development which include vaccine antigens, enzymes, cytokines, monoclonal antibodies and their fragments and a few are approved [119,120]. The approval of the first PMP in the commercial market and promising results of plant-proteins in clinical trials pave the way for the further development of recombinant products.

Several examples of such PMPs and the detailed advantages, limitations and challenges of plant expression system for the production of the desired targets have been comprehensively reviewed elsewhere [121,122,123,124,125,126,127,128]. The different plant-based expression systems range from transgenic plants to cell suspensions cultures are available for AMP production in plants [129,130,131,132,133] which are described briefly in the following sections.

## 5. Strategies for Protein Production in Plants

**Figure 1:**
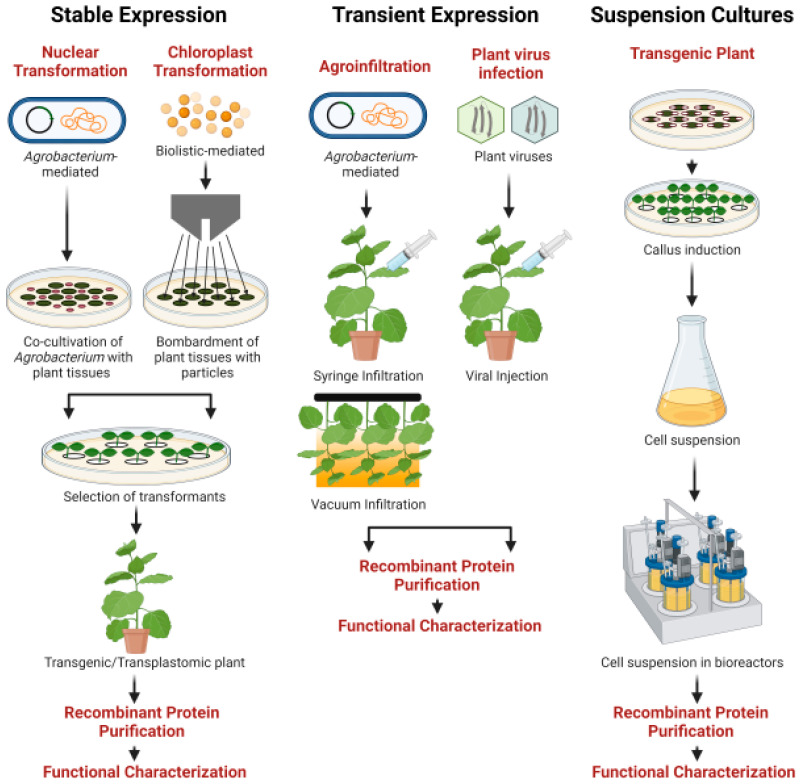
Schematic representation of technologies available in plant-based platforms for the production of recombinant biopharmaceuticals. During initial stages of plant molecular farming, recombinant protein expression in plants were based on stable expression in which the gene of interest is cloned into plant expression vector and transformed into plant nucleus/chloroplast either by *Agrobacterium* or biolistic mediated method to produce transgenic or transplastomic plants. Transient expression combines the advantages of *A. tumefaciens* and plant viral elements for the rapid and high yield protein production. Genetically modified suspension-cultured plant cells integrate the advantages of whole-plant systems with those of bacterial fermentation and mammalian cell cultures. Suspension cultures can be grown in bioreactors under controlled environment for recombinant protein production.

The production of recombinant proteins in plants form an ideal cost-effective platform gaining attraction for commercial biopharmaceutical production. The strategies employed for recombinant protein production in plants are stable expression, transient expression and suspension cell cultures (Figure 1). The stable expression is a conventional method of recombinant technology which involves the incorporation of foreign genes into nucleus for nuclear genomic expression [134] and/or to chloroplast for plastid genomic expression [135] resulting in the generation of stable transgenic/transplastomic plant lines.

### 5.1. Stable Nuclear Expression

Nuclear expression facilitates the stable integration of transgenes into the nuclear genome of plant cells. This technique is regulated by transcription of the gene of interest in the nucleus and then translation in the cytoplasm [136]. The most widely used gene delivery system into the plant is via *Agrobacterium tumefaciens*-mediated transformation. The plant bacterium, *A. tumefaciens*, has the capability to deliver a particular DNA segment (T-DNA) into the plant nucleus which is commonly localized on the tumor-inducing (Ti) plasmid of *Agrobacterium* [137]. Meanwhile, plant transformation utilizes a binary vector system devised according to the T-DNA of *Agrobacterium*. Consequently, the T-DNA-containing gene expression cassette is separated from the vector backbone and transformed into plants, permitting easy genetic engineering of plants. This vector system is introduced into a modified *Agrobacterium* (not containing bacterial genes within the T-DNA region) to infect the plant cells or tissues and transfer the T-DNA-containing gene of interest from the binary vector for expression in nuclear genome of plant host [138]. These tissues are cultivated in an antibiotic-containing growth medium, allowing selective growth of transformants harboring the gene of interest. Then, growth of callus tissue and development of shoots and roots are observed. Upon successful rooting, plantlets are transferred to the soil and the expression of foreign genes in the transgenic lines can be characterized [139]. The *Agrobacterium*-mediated transformation offers the simplest and a conventional method for genetic modification of crops with the horizontal transgene transfer and consistent recombinant protein expression [140]. However, few associated disadvantages of this method includes gene silencing, transgene contamination risk, potential interactions with natural products, low yields (about <1% of total soluble protein) and time-consuming genetic manipulation [136,141,142]. An early study of pharmaceutical recombinant antibodies stably integrated and produced in transgenic tobacco plants was recorded in 1989. Since then, several proteins have been produced in stably transformed plants, including anti-cancer agents [143,144] and antimicrobial peptides as anti-infectives [145]. AMPs with anti-bacterial or anti-fungal properties can be stably expressed in plants which confers disease resistance against plant pathogens which in turn increases the yield, quality and safety of agricultural products.

### 5.2. Stable Chloroplast Expression

Chloroplast expression directs incorporation of transgenes into the chloroplast genome of plant cells. This approach effectively transforms foreign genes into plant chloroplasts by using a particle gun or gene gun or biolistic transformation method. In particular, plant tissues are bombarded with DNA-coated gold or tungsten particles [146]. Then, plant tissues are cultivated in suitable growth medium supplemented with appropriate antibiotics, which confer selection of transformants containing the gene of interest. Similar like nuclear transformation, characterization of callus formation, shoots and roots development is observed. The young plantlets are transplanted into soil to generate mature transplastomic plants. Chloroplast transformation of recombinant gene offers several advantages compared to nuclear transformation. The chloroplast genome provides ease of manipulation as DNA-containing cassette can be inserted in between functional chloroplast genes by homologous recombination [147]. The specific targeting ensures high levels of expression and prevents gene placement into a poorly transcribed region of the genome. Due to high copies of chloroplast in plant cells, optimal yields of recombinant proteins have been attained by chloroplast expression [136,147]. Additional advantages include no risk of transgene contamination or leakage into the environment since chloroplast genes are maternally inherited [146], a neutral pH and low number of active proteases [148]. The expression of therapeutic proteins in chloroplast has been well-explored for several antigens, growth factors, interferons and many other pharmaceutical proteins [140,149,150,151].

### 5.3. Transient Expression

Transient gene expression involves rapid production of recombinant proteins without chromosomal integration into the plant cell genome. It has been employed as an approach for determining the expression efficiency of transgenes in the plant nucleus after a short period of time [152]. There are two promising methods of transient expression involving plant pathogen vectors, namely, plant viruses (plant viral infection) and *A. tumefaciens* (agroinfiltration). Plant virus-mediated transient expression directs amplification of viral vectors within plant cells and introduces the transgene of interest by utilizing mature viral particles [153]. Examples of plant virus-based vectors include but not restricted to tobamoviruses, potexviruses, potyviruses, bromoviruses, comoviruses, and geminiviruses [154,155,156]. This strategy has shown advantages and suitability to produce several plant-based biopharmaceuticals [157]. In addition, they have been characterized for their fast transmission from one plant cell to another, yielding high expression efficiency [158]. Meanwhile, risk of viral vector contamination in plants and the environment has to be regarded with careful consideration [159].

*Agrobacterium*-mediated transient expression refers to the process of infecting plant leaves with *Agrobacterium* cells suspension containing T-DNAs with gene of interest [160]. It can be performed either by infiltration using syringe without a needle (syringe infiltration) or by large-scale infiltration (vacuum infiltration). *Agrobacterium* infection spreads across the site of injection and infected plants can be harvested within few days of post-infiltration. Transient approach is the method of choice for the scalable production of AMPs for large scale applications in the food industry (as preservative), as topical disinfectant or as a feed supplement for livestock or poultry [161].

### 5.4. Suspension Cultures

Suspension plant cell cultures are more promising and ideal platforms than using the whole plants to produce various important biological active products. These cultures are grown in controlled environments under monitoring and defined conditions for the growth of plant cells thus complying all the regulatory concern. The plant cells are cultured in aseptic in vitro growth conditions in a sterile sealed container without any human or microbial contaminants [162,163]. Other biosafety and environmental issues can also be overcome by using plant bioreactors preventing cross fertilization and transmission of pollen. The production costs for recombinant proteins using plant suspension cell cultures are quite low in comparison to mammalian and bacterial systems as they require simple growth media and nutritional requirements [164]. The downstream purification and processing of plant produced products does not require any complex methods [165]. The first FDA-approved plant-produced pharmaceutical taliglucerase-α was produced in carrot suspension cell cultures almost reducing the conventional orphan drug treatment costs by 75% [166]. Other popular plant cells include BY-2 and NT-1 tobacco strains that are used as bioreactors where the proteins can be secreted into the culture medium simplifying the purification process. BY-2 cell cultures were used to produce human monoclonal antibody M12 in a 200 L bioreactor yielding 20 mg/L of the mAb [164,167]. The BY-2 cell lines have the capacity to multiply up to 100-fold in one week with a generation time of 16–24 h under defined growth parameters [168]. Suspension cultures hold significant potential in therapeutic AMP production for medical applications as they are easy to scale-up, compliant with GMP and they meet regulatory requirements for biopharmaceutical production.

## 6. AMP Expression in Plants

Several AMPs have been expressed in plants with the perspective of clinical and agricultural development. Plants have come into limelight for the expression of AMPs in desired crop plants for direct defense against pathogens and also large-scale and cost-effective production of recombinant AMPs. Although protein accumulation varies between the AMP expressed, but the functional activity of the recombinant AMP confirmed its active form. Protegrin-1, a broad-spectrum AMP was expressed in low alkaloid tobacco species using transient approach and was found to be effective against *K. pneumoniae*, *S. aureus*, *E. coli*, *M. bovis* BCG, and *C. albicans* [169]. Lfchimera, a chimerical peptide was codon optimized, expressed in plant culture system and tobacco hairy roots in vitro and significant antimicrobial activity was reported against clinical and phytopathogenic bacteria [170,171]. AMPs apart from exhibiting antimicrobial activity, they were further investigated for inducing resistance against various bacterial and fungal pathogens in plants [162]. The peptide LL-37 was produced in transgenic barley by expressing codon optimized chimeric LL-37 under the influence of endosperm specific promoter of barley B1 hordein gene, accumulating upto 0.55 mg/kg of grain and the plant-produced LL-37 was biologically active [172]. An insect antimicrobial peptide, thanatin S, was expressed by fusing with signal peptide of rice Cht1 in *Arabidopsis*, that showed enhanced resistance to phytopathogenic fungi and bacteria [173]. In a study by Jung et al., human cathelicidin hCAP18 was expressed in Chinese cabbage fusing the DNA encoding fragment for this AMP with C-terminal end of endopolygalacturonase inhibiting protein under the control of CaMV 35 S promoter showing varied levels of resistance to bacterial and fungal pathogens [174]. Two proteins, snakin-2 (SN2) a cysteine-rich peptide and extensin-like protein (ELP) a major cell-wall hydroxyproline-rich glycoprotein were over expressed in tomato cultivars and showed resistance against *Clavibacter michiganensis* subsp. *michiganensis (Cmm)* [175]. A snakin-1 gene isolated from potato was found to have in vitro antimicrobial activity and when transformed into wheat by particle bombardment, showed effective protection against soil borne fungus *Gaeumannomyces graminis* var. *tritici* which causes root disease [176]. Defensins, SmAMP 2 gene, sarcotoxin IA, retrocyclin 101, hevein like peptides, C4V3, trichokonins, cecropin B, temporin A, snakin-2, cathelicidins and MsrA2 were also expressed in different plant species as listed in Table 3.

## 7. Conclusions

Diverse AMPs hold major potential for the development of innovative approaches in both clinical and agricultural biotechnology. Disease-resistant plant traits developed by introducing AMPs might increase yields and offer safety of agricultural products against phytopathogens. Further recombinant expression of AMPs in plant platforms overcome the limitations associated with the large-scale production of these recombinant peptides for clinical use. The urgent need of rapid, cost-effective protein production systems for the production of large amounts of recombinant protein has been driving the plant molecular farming research. There has been much progress in our understanding of this field and extensive research has been performed over the last three decades on plant-based biopharmaceutical production against various pathogens. The plant-derived proteins are shown to be functional and even shown to be effective in clinical trials. However, plant-made pharmaceuticals still encounter some technological and regulatory issues limiting prospective investors eventually resulting in a long timeframe of potential products from bench-to-market. Despite the many proof-of-concept studies, few products are approved for commercial applications. The challenges faced by PMPs during initial stages of plant molecular farming such as longer production time, transgene escape and safety have been addressed in recent decades. The issues related to low yield and time associated with the stable expression have been addressed by developing transient expression systems. Thus, the proper selection of expression strategy, vector, and extraction/purification techniques is essential to achieve high product yield, desired functionality, safety and quality of the products. Furthermore, the recent advances in the plant biotechnology have pushed various regulatory bodies to develop regulatory frameworks for the process of genetic transformations or on the final plant-derived product [208]. There is substantial evidence showing the capability of making proteins with high quality to address a range of human health-related issues particularly in low-income and middle-income countries. The FDA approval for the therapeutic enzyme Elelyso was a major milestone in the field. Most likely, we can expect a significant number of plant-derived biopharmaceuticals on the market in the upcoming years. There are several promising AMPs which are in different stages of clinical trials. These AMPs could be potential candidates for plant-based manufacturing. Thus, the integration of our existing knowledge of the plant biotechnology, huge strides that have been made in plant transient expression and glycoengineering strategies coupled with the design, development and accessibility of AMPs could make an ideal foundation for the design of a novel class of plant-derived AMP based therapeutics that hold promising potential. In summary, biotechnological perspectives for the rapid large-scale production of AMPs in plant systems has been provided. The existing knowledge on plant expression system opens the way to produce and evaluate the potentiality of AMPs that could be rapidly manufactured, at low cost and with negligible risk, to fight against drug resistant pathogens in post-antibiotic era. Altogether production of AMPs in plants is considered a prospective tool for novel applications in medicine and agriculture.

## Figures and Tables

**Table 2:** Advantages and disadvantages of different host systems available for the production of heterologous proteins for pharmaceutical applications (adapted from Shanmugaraj et al. [114]).

Expression System	Advantages	Disadvantages
Bacteria	Easy to manipulate Low cost High expression Ease of scale up Short turnaround time Established regulatory procedures and approval	Improper folding Lack of post-translational modifications, which may affect the protein function. Endotoxin accumulation
Mammalian Cells	Proper folding and authentic post-translational modifications Existing regulatory approval	High production cost Expensive media and culture condition requirements
Yeast	Rapid growth and scalable Easy to manipulate Simple and inexpensive media requirements and culture conditions Post-translational modifications of recombinant proteins	Difficulty in cell disruption due to the thick and hard cell walls Hyperglycosylation of proteins
Insect cells	High expression levels Ability to produce complex proteins including secreted, membrane and intracellular proteins Proper folding and post-translational modifications	High cost and time consuming Expensive media and culture condition requirements
Plants	Rapid and affordable Optimized growth conditions Free from pathogen and bacterial toxin contaminants Economical Post-translational modification somewhat similar like mammalian system	Regulatory compliance Limited glycosylation capacity

**Table 3:** Some of the candidate AMPs expressed in plant hosts.

Anti-Microbial Peptide	Plant Species	Stable or Transient	Nucleus or Chloroplast	Expression Level	Application	Reference
MSI-99 (Magainin)	Tobacco (*Nicotiana tabacum*)	Stable	Chloroplast	Undefined	Enhanced resistance to phytopathogenic bacteria (*Pseudomonas syringae*) and fungi (*Aspergillus flavus*; *Fusarium moniliforme*; *Verticillium dahlia*)	[177]
MsrA2 (Dermaseptin)	Potato (*Solanum tuberosum*)	Stable	Nucleus	1–5 μg/g FW	Broad-range and enhanced resistance to virulent phytopathogenic fungi (*Alternaria*, *Cercospora*, *Fusarium, Phytophthora*, *Pythium, Rhizoctonia; Verticillium* sp.) and bacteria (*Erwinia carotovora*)	[178]
	Tobacco (*Nicotiana tabacum*)	Stable	Nucleus	6–7 μg/g FW	Resistance to phytopathogenic fungi (*Fusarium solani; F. oxysporum; Alternaria alternata; Botrytis cinerea; Sclerotinia sclerotiorum*), oomycete (*Pythium aphanidermatum*) and bacterium (*Pectobacterium carotovorum*)	[179]
Thi2.1 (Thionin)	Tomato (*Lycopersicon esculentum*)	Stable	Nucleus	Undefined	Crop protection (*F. oxysporum* f. sp. *lycopersici; R. solanacearum* strain Pss4)	[180]
Mj-AMP2 (Knottin)	Rice (*Oryza sativa*)	Stable	Nucleus	0.32–0.38% total protein	Enhanced resistance to fungal pathogen (*Magnaporthe oryzae*)	[181]
ChIFN-alpha (interferon-α)	Lettuce (*Lactuca sativa*)	Transient	Nucleus	0.393 μg/kg FW	Antiviral activity against vesicular stomatitis virus (VSV)	[182]
Lipid Transfer Proteins (LTPs)	Tobacco (*Nicotiana tabacum*)	Stable	Nucleus	Undefined	Enhanced resistance to pathogen (*Phytophthora nicotianae; Pseudomonas syringae* pv. *tabaci*)	[183]
*Dm*-AMP1 (Defensin)	Rice (*Oryza sativa*)	Stable	Nucleus	0.43–0.57% total soluble protein	Enhanced resistance to pathogen (*Magnaporthe* oryzae; *Rhizoctonia solani*)	[184]
rLF (Lactoferrin)	Rice (*Oryza sativa*)	Stable	Nucleus	0.1% rice bran weight	Functional feed additive on early weaned piglets	[185]
Rs-AFP2 (Defensin)	Rice (*Oryza sativa*)	Stable	Nucleus	0.45–0.53% total soluble protein	Enhanced resistance to fungal pathogen (*Magnaporthe oryzae; Rhizoctonia solani*)	[186]
CecB (Cecropin)	Tomato (*Solanum lycopersicum*)	Stable	Nucleus	0.001 µg/mg FW	Plant protection against bacterial pathogens (*Ralstonia solanacearum*; *Xanthomonas campestris*)	[187]
Retrocyclin-101 (Defensin)	Tobacco (*Nicotiana tabacum*)	Stable	Chloroplast	32–38% total soluble protein	Control viral (tobacco mosaic virus) and bacterial (*Erwinia carotovora*) infections	[188]
Protegrin-1 (Cathelicidin)	Tobacco (*Nicotiana tabacum*)	Stable	Chloroplast	17–26% total soluble protein	Control bacterial infections (*Erwinia carotovora*)	[188]
	Tobacco (*Nicotiana tabacum*)	Transient	Nucleus	Undefined	Control mammalian bacteria (*Klebsiella pneumoniae*; Staphylococcus *aureus*; *Escherichia coli*; *Mycobacterium bovis*) and fungal (*Candida albicans*) pathogens	[169]
*Petunia* Floral defensins	Banana (*Musa* spp.)	Stable	Nucleus	Undefined	Effective resistance against pathogenic fungal *Fusarium oxysporum* f. sp. *cubense* (foc) infection	[189]
Snakin-2 (Snakin)	Tomato (*Solanum lycopersicum*)	Stable	Nucleus	Undefined	Enhanced resistance to *Clavibacter michiganensis* subsp. *michiganensis*	[175]
Lactoferricin B (Lactoferrin)	Tobacco (*Nicotiana tabacum*)	Stable	Nucleus	Undefined	Enhanced tolerance to pathogenic bacterial (*Pseudomonas syringae* pv. *tabaci*) and fungal (*Botrytis cinerea*) diseases	[190]
PmAMP1 (cysteine-rich protein)	Canola (*Brassica napus*)	Stable	Nucleus	Undefined	Effective resistance against fungal pathogens (*Alternaria brassicae*; *Leptosphaeria maculans*; *Sclerotinia sclerotiorum*)	[191]
hCAP18/LL-37 (Fusion of two cathelicidin antimicrobial proteins)	Chinese cabbage (*Brassica rapa* cv. Osome)	Stable	Nucleus	Undefined	Enhanced resistance to bacteria (*P. carotovorum* subsp. *carotovorum*) and fungal (*Fusarium oxysporum* f. sp. *Lycopersici*; *Colletotrichum higginsianum*; *Rhizoctonia solani*	[174]
Lactostatin (anionic peptide)	Rice (*Oryza sativa*)	Stable	Nucleus	2 mg/g dry seeds	Anti-hypercholestero lemic drug for potential clinical use	[192]
SP1-1 (*de*-*novo* designed)	Tobacco (*Nicotiana benthamiana*)	Transient	Nucleus	0.025 mg/g FW	Antimicrobial activity (*P. syringae pv. Syringae*; *P. syringae pv. Tomato*; *P. corrugate*; *Pectobacterium carotovorum* ssp. *carotovorum*)	[193]
SN-1 (Snakin)	Wheat (*Triticum aestivum*)	Stable	Nucleus	Undefined	Antifungal activity in vitro and enhanced resistance to fungus (*Gaeumannomyces graminis* var. *tritici*) and	[176]
Thanatin (S) (synthetic thanatin)	Arabidopsis (*Arabidopsis thaliana*)	Stable	Nucleus	Undefined	Acquired resistance to bacterial pathogen (*Pseudomonas syringae* pv. *tomato*.) and fungal pathogens (*Botrytis cinerea*; powdery mildew) Antibacterial and antifungal activity *in vitro*	[173]
LL-37 (Cathelicidin)	Tomato (*Solanum lycopersicum*)	Stable	Nucleus	16.8–58.2 µg/mL total soluble protein	Enhanced antibacterial activity (*Pectobacterium carotovorum* ssp. *Carotovorum* (Pcc); *Xanthomonas campestris* pv. *Vesicatoria* (Xcv)	[194]
	Barley (*Hordeum vulgare* L.)	Stable	Nucleus	0.55 mg/kg seeds	Antibacterial activity against *E. coli* TOP10 *in vitro*	[172]
BP100.gtag (synthetic peptide)	Rice (*Oryza sativa*)	Stable	Nucleus	0.5% total soluble protein	Plant protection against bacterial pathogens (*Erwinia amylovora*; *Pseudomonas syringae*; *Xanthomonas axonopodis*)	[195]
CecA (Cecropin)	Rice (*Oryza sativa*)	Stable	Nucleus	1–4 μg/g seeds	Resistance to fungal pathogen (*Fusarium verticillioides*) and bacterial pathogen (*Dickeya dadantii*)	[196]
Recombinant colicins (Colicin)	Tobacco (*Nicotiana benthamiana*)	Transient	Nucleus	0.6–3 mg/g FW	Effective and broad control of foodborne pathogenic *Escherichia coli* strains	[197]
	Tobacco (*Nicotiana benthamiana*)	Transient	Nucleus	0.58–2.31 mg/g FW	Broad activity, high potency, and purity as food antibacterial	[198]
Retrocyclin	Tobacco	Stable	Chloroplast	116 μg of RC101/g of lyophilized leaf	Effective against *Streptococcus mutans* and impaired biofilm formation following a single topical application of tooth-mimetic surface.	[199]
Protegrin	Tobacco	Stable	Chloroplast	Undefined	Effective against *Streptococcus mutans* and impaired biofilm formation following a single topical application of tooth-mimetic surface.	[199]
pro-SmAMP2 (Hevein-like peptide)	Potato (*Solanum tuberosum*)	Stable	Nucleus	Undefined	Crop protection from *Alternaria* sp. and *Fusarium* sp. pathogens in resistant potato cultivar	[200]
D2A21 (synthetic peptide)	Citrus fruit (*Carrizo citrange*)	Stable	Nucleus	Undefined	Reduced development of canker disease caused by bacterium (*Xanthomonas citri*)	[201]
PaeM4 (Pyocin)	Tobacco (*Nicotiana benthamiana*)	Transient	Nucleus	800 μg/g FW	Broad spectrum of antimicrobial activity against clinical isolates of *Pseudomonas aeruginosa*	[202]
CBD-alfAFP (Defensin)	Tobacco (*Nicotiana tabacum*)	Stable	Nucleus	Undefined	Enhanced resistance to plant pathogen (*Fusarium solani*)	[203]
LFchimera (Lactoferrin-derived peptides)	Tobacco (*Nicotiana tabacum*)	Stable	Nucleus	Undefined	Antimicrobial activity against clinical (*Escherichia coli*; *Staphylococcus aureus*) and phytopathogenic bacteria (*Ralstonia solanacearum*; *Erwinia amylovira*)	[171]
	Tobacco (*Nicotiana tabacum*)	Suspension Cultures	Hairy roots	4.8 μg/g FW	Effective antimicrobial activity against *Escherichia coli*	[170]
*Penicillium digitatum* AfpB (antifungal protein)	Tobacco (*Nicotiana benthamiana*)	Transient	Nucleus	225 ± 37 µg/g FW	Protect tomato plants against *Botrytis cinerea* causing grey mold disease	[204]
DrsB1 (Dermaseptin)	Tobacco (*Nicotiana tabacum*)	Suspension culture	Nucleus	Undefined	Effective antimicrobial effects of plant bacterial and fungal phytopathogens	[205]
	Tobacco (*Nicotiana tabacum*)	Stable	Nucleus	5.5–6.0 µg/g FW	Enhanced resistance to plant pathogens (*Alternaria alternata*; *Alternaria solani*;, *Fusarium oxysporum*; *Fusarium solani* fungi)	[206]
Laterosporulin-1 (synthetic anionic AMP/ELP fusion)	Tobacco (*Nicotiana benthamiana*)	Transient	Nucleus	375 µg/g FW	High antibacterial activity against *Staphylococcus epidermidis*	[161]
ADP2-3 (synthetic anionic AMP/ELP fusion)	Tobacco (*Nicotiana benthamiana*)	Transient	Nucleus	563 µg/g FW	High antibacterial activity against *Staphylococcus epidermidis*	[161]
Colicin M (Colicin)	Tobacco (*Nicotiana tabacum*)	Stable	Nucleus	2 mg/g FW	Antibacterial activity against control and clinical pathogens (*Escherichia coli*; *Klebsiella pneumoniae*)	[207]

## Data Availability

No new data were created or analyzed in this study. Data sharing is not applicable to this article.
